# Biosurfactant production by *Bacillus subtilis* SL and its potential for enhanced oil recovery in low permeability reservoirs

**DOI:** 10.1038/s41598-022-12025-7

**Published:** 2022-05-11

**Authors:** Bo Wu, Jianlong Xiu, Li Yu, Lixin Huang, Lina Yi, Yuandong Ma

**Affiliations:** 1 University of Chinese Academy of Sciences, Beijing, China; 2 Institute of Porous Flow and Fluid Mechanics, University of Chinese Academy of Sciences, Hebei, China; 3 PetroChina Research Institute of Petroleum Exploration and Development, Beijing, China

**Keywords:** Biotechnology, Energy science and technology

## Abstract

Microbial enhanced oil recovery (MEOR) technology is an environmental-friendly EOR method that utilizes the microorganisms and their metabolites to recover the crude oil from reservoirs. This study aims to research the potential application of strain SL in low permeability reservoirs. Strain SL is identified as *Bacillus subtilis* by molecular methods. Based on the mass spectrometry, the biosurfactant produced by strain SL is characterized as lipopeptide, and the molecular weight of surfactin is 1044, 1058, 1072, 1084 Da. Strain SL produces 1320 mg/L of biosurfactant with sucrose as the sole carbon source after 72 h. With the production of biosurfactant, the surface tension of cell-free broth considerably decreases to 25.65 ± 0.64 mN/m and the interfacial tension against crude oil reaches 0.95 ± 0.22 mN/m. The biosurfactant exhibits excellent emulsification with crude oil, kerosene, octane and hexadecane. In addition, the biosurfactant possesses splendid surface activity at pH 5.0–12.0 and NaCl concentration of 10.0% (w/v), even at high temperature of 120 °C. The fermentation solution of strain SL is applied in core flooding experiments under reservoir conditions and obtains additional 5.66% of crude oil. Hence, the presented strain has tremendous potential for enhancing the oil recovery from low-permeability reservoirs.

## Introduction

Low permeability reservoirs, generally characterized with the permeability less than 50 mD (In China), are difficult to generate economic volume of petroleum by conventional oil recovery technologies^1,2^. Low permeability reservoir primarily possesses the properties of small pores, fine throats, high filtration resistance, low oil productivity and low water absorbing capacity^3,4^. In recent years, with the consumption of conventional oil and gas resources, the efficient exploitation of low permeability reservoir has become important and difficult in the oil industry^5^. Hydraulic fracturing and CO_2_ flooding are primary methods to explore oil in low permeability reservoirs^6^. However, those two methods possess both merits and defects that have around the interest of many scholars. Hydraulic fracturing occupies a critical position in the development of oil production, but it brings about detrimental pollution to the water environment. Likewise, CO_2_ flooding has indeed enhanced the oil recovery but elevates the mining costs. Therefore, as people gradually awaken to the awareness of environmental protection, how to extract the residual oil with green techniques after water/gas flooding is becoming a hotspot at present.

Microbial enhanced oil recovery (MEOR) is an environment-friendly tertiary oil recovery method that can improve the physicochemical properties of residual oil and reservoirs to extend the production life of oil wells and reduce the expenses. MEOR mainly comprises the application of microorganisms and their metabolic products including biosurfactants, biopolymers, biomass, biogas and acids. In addition, many dominant strains can be widely used in MOER process due to their capability of oil degradation and/or emulsion, such as *Bacillus* sp., *Pseudomonas* sp., *Saccharomyces* sp., *Rhodococcus* sp., *Acinetobacter* sp. However, so far, it should be noted that the the application of these bacteria in MEOR is primarily concentrated on reservoirs with medium–high permeability, rarely referring to those with low permeability. Xiao et al. studied the application potential of indigenous bacteria in enhancing oil recovery of low permeability reservoirs^7^. They stated that the indigenous bacteria stimulated by activator can generate surface active substance to transform the wettability of rock surface from oil-wet to water-wet. Similarly, Ghojavand et al. selected the biosurfactant produced by *Bacillus mojavensis* (PTCC 1696) to extract residual oil from low permeability carbonate reservoirs and achieved satisfactory results^8^. Thus, it is not difficult to see, the surfactants produced by strains play an important role in the enhanced oil recovery of low permeability reservoirs. Biosurfactant, produced by microbial metabolism, is a kind of amphoteric compound that contains hydrophilic group and lipophilic group^9^. Biosurfactant can not only reduce the oil–water interfacial tension, and alter the rock wettability, but also accelerate the emulsification of crude oil. Based on the extensive reviews, it has been demonstrated that the characteristics of biosurfactant depend largely on its molecular mass. The low molecular mass surfactants, like lipopeptide biosurfactants, are normally associated with the wettability alteration and reduction of ITF^10,11^ whereas the high molecular mass surfactants are always characterized with splendid emulsifying capacity^12^. Moreover, biosurfactant is of some special advantages over chemical surfactant, such as the environmental compatibility and wide application prospect.

The aim of this study is to evaluate the feasibility of strain SL in low permeability reservoirs. Firstly, the strain SL was identified by morphological observation and 16S rDNA sequence analysis. The microbial growth of each stage was monitored by plate count. Then, the biosurfactant produced by strain SL was obtained by acid precipitation and solvent extraction method to estimate its emulsifying capacity, salt tolerance, temperature resistance and pH-hardy. Ultimately, the enhanced oil recovery in low-permeability reservoirs by strain SL was deeply investigated and discussed.

## Material and methods

**Figure 1:**
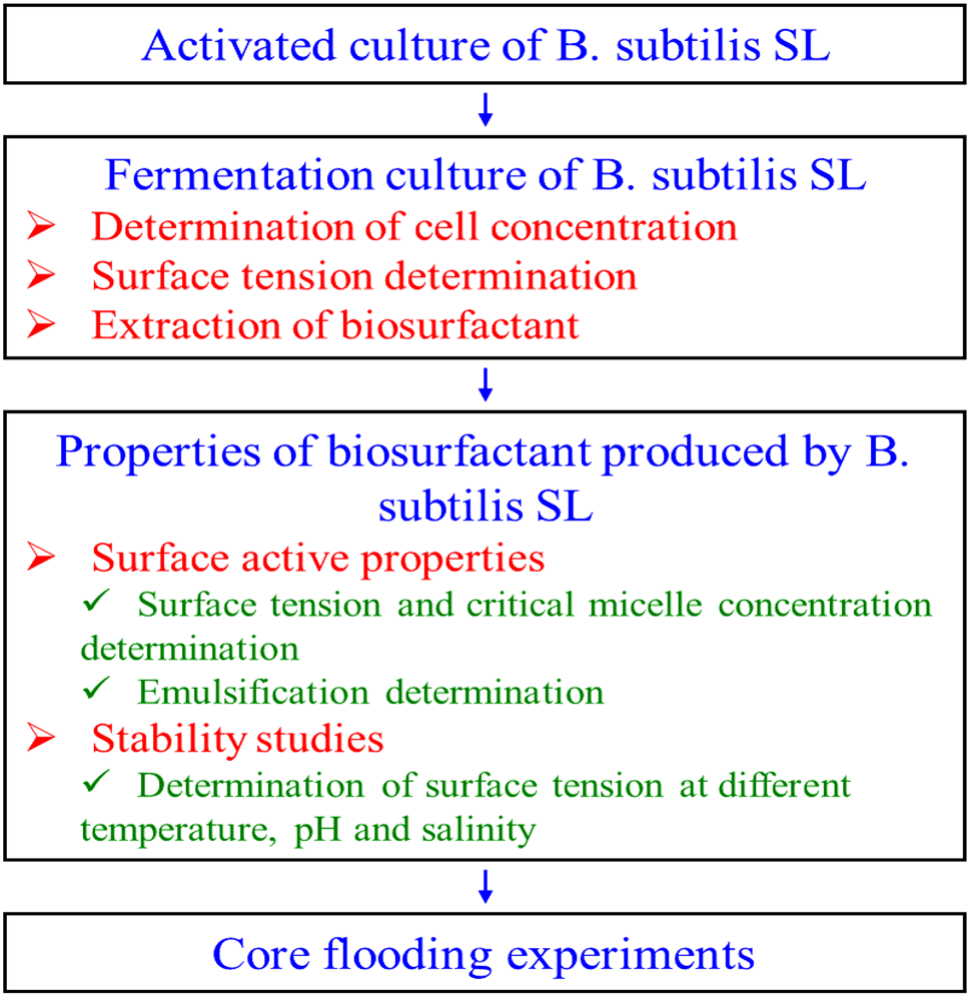
Research method flowchart.

Materials and methods are described in this section, and the experimental procedure is presented in Fig. 1.

### Crude oil and culture media

The crude oil and formation water in this study were acquired from Daqing Oilfield Branch of China National Petroleum Corporation. All the chemicals, analytical grade or better, were purchased from Sinopharm Chemical Reagent Co., Ltd., China.

A series of culture media were prepared to satisfy the experimental demands: the Luria–Bertani (LB) medium containing 5.0 g/L yeast extract, 10.0 g/L NaCl, 10.0 g/L peptone; the LB solid medium with 2.0% agar ; the biosurfactants production medium containing 30.0 g/L sucrose, 3.0 g/L NaNO_3_, 4.0 g/L K_2_HPO_4_·H_2_O, 3.4 g/L KH_2_PO_4_, 0.6 g/L MgSO_4_·7H_2_O, 1.2 g/L yeast extract. Moreover, the pH of involved medias was adjusted to 7.0 via HCl (1.0 mol/L) and NaOH (1.0 mol/L).

### Microorganisms and biosurfactant preparation

Taken from the strain library in the Microbiology Laboratory of Institute of Porous Flow and Fluid Mechanics, the strain SL was selected to produce surfactants and its Genomic DNA was extracted using TIANamp Bacteria DNA Kit (Tiangen Biotech Co., Ltd, Beijing, China). The 16 S rDNA of strain SL was amplified by means of universal PCR primers pair 27 F (5-AGAGTTTGATCCTGGCTCAG-3) and 1541 R (5-AAGGAGGTGATCCAGCC-3). The reaction process was proceeded in sequence as follows: 5 min at 94 °C, 30 cycles of 45 s at 94 °C, 45 s at 55 °C and 2 min at 72 °C, and a final extension step of 10 min at 72 °C. The obtained gene sequences were compared with the closest strains in the GenBank database using the Basic Local Alignment Search Tool (BLAST) in term of percent identity. The sequences were aligned by Clustal X version 2.0 software and the phylogenetic tree was reconstructed using MEGA version 6.0 software, and inferred by using the neighbor-joining method with 1000 replicates of bootstrap values.

LB medium was used for the preparation of seed broth. The culture was cultivated in an orbital shaker with 180 rpm for 18 h and the operating temperature was set to 37 °C. Afterwards, Erlenmeyer flasks filled with 400 mL of production medium and 2.0%(v/v) seed broth were incubated in the shaker (37 °C, 180 rpm and 96 h). It’s noted that all the mediums were sterilized by autoclaving at 121 °C for 15 min, before adding seed solution. The samples were harvested every few hours until approaching 72 h and analyzed for cell concentration, biosurfactant concentration, and surface tension. Significantly, the measurement of biosurfactant concentration and surface tension (SF) were used by cell-free supernatant. The biosurfactant concentration was indirectly quantified by oil spreading method^13^, in which the oil was mixed by liquid paraffin and Sudan IV.

### Measurement of cell concentration

Cell enumeration in terms of colony forming units (CFU) was performed by the gradient dilution coating method. The nutrient agar plates were prepared by pouring 25 ml of sterilized media aseptically in each sterilized plate. The samples were collected at specific time and diluted by distilled water. Subsequently, 100 μL drops of samples possessing different dilutions were dipped in the prepared nutrient agar plate and incubated for 24 h at 37 °C. Colony forming units were counted and multiplied with the relative dilution factor to gain the CFU values. This process was repeated three times to obtain the average value of cell concentration, minimizing the measurement error of test samples.

### Biosurfactant extraction and mass spectrometry analysis

In this part, the acid precipitation and solvent extraction experiments were carried out to extract the biosurfactants from cell-free supernatant ^14,15^. Bacterial cells were separated from the production medium by centrifuging at 10,000 rpm for 20 min at 4 °C (Beckman Coulter, JLA-16.250 rotor, USA). The cell-free supernatant was acidified to pH 2.0 using 6 M HCl and kept overnight at 4 ℃ to collect the primary precipitate biosurfactant. After centrifugation at 5000 rpm and 4 °C for 30 min, the supernatant was removed and the precipitates were dissolved in pure methanol. The undissolved fractions were separated by filtration. The methanolic extracts were evaporated to dryness under vacuum by a rotary evaporator^16^. The extracted biosurfactant was dissolved in methanol and subjected to mass spectrometry characterization. The electrospray ionization mass spectrometry (ESI–MS) analysis was performed according to the method reported before^17^.

### Measurement of surface activity

Surface tension (SF) of the test sample was measured by a surface tensiometer FTA1000B (First Ten Angstroms, Portsmouth, Virginia, US). For the calibration of the instrument, the SF of distilled water was measured and then repeated three times to obtain the average value, effectively characterizing the surface activity of the sample. The biosurfactant concentration expressed in terms of critical micelle concentration (CMC) was estimated by measuring the SF of crude oil with varied dilutions. Meanwhile, a interfacial tensiometer (TX-500C, The Rationale Electronics Co., Ltd. Shanghai, China) was employed to determine the interfacial tension (IFT) between the and supernatant solution and crude oil, among which the crude oil was derived from the of Daqing Oilfield with density of 0.8737 g/cm^3^ and viscosity of 17.2 mPa·s. All the measurements were accomplished in triplicate and the corresponding results were calculated as mean ± standard deviation (s.d.) values.

### Determination of the emulsification index (E_24_)

Emulsifying activity of the biosurfactant produced by strain SL was determined by measurement of the emulsion index (E_24_). The cell-free broth was mixed with crude oil, kerosene, octane and hexadecane in equal volume, respectively, and then vortexed for 2 min^12^. The sample was allowed to stand for 24 h at room temperature. As shown in Eq. (1), the emulsification index (E_24_) was calculated by dividing the height of emulsion layer to the total height of the liquid column. Likewise, all the measurements in this part were manipulated in triplicate at ambient temperature and the results were calculated as mean ± standard deviation (s.d.) values.

1 $$Emulsification\ Index(E_{24} ) = \frac{Height\ of\ the\ emulsion\ layer}{{Total\ height\ of\ the\ liquid\ column }} \times 100\%$$

### Stability studies

The stability of biosurfactants produced by strain SL was evaluated under a wide range of temperature, pH, and salinity. Specially, the aliquots of cell-free supernatants (10 mL) were incubated at different temperatures (4 °C, 37 °C, 50 °C, 60 °C, 100 °C) for 24 h and cooled at room temperature. By contrast, another cell-free supernatant (10 mL) was subjected to autoclavation at 120 °C for 15 min and cooled at room temperature. To study the effect of pH, the aliquots were adjusted to 2.0, 4.0, 6.0, 8.0, 10.0 with HCl (1.0 mol/L) /NaOH (1.0 mol/L), placed in an incubator for 1 h at 37 °C and cooled at room temperature. Similarly, the aliquots were added with varied concentrations (%, w/v) of NaCl (0, 0.5, 1.0, 2.0, 3.0, 4.0, 5.0, 6.0, 7.0, 8.0, 9.0, 10.0, 12.0, 14.0, 16.0, 18.0, 20.0) to investigate the effect of salinity on biosurfactant activity and the laboratory conditions were identical with pH experiments. The surface tension of all treated samples was measured by a surface tensiometer FTA1000B (First Ten Angstroms, Portsmouth, Virginia, US). This process was repeated three times and the results were calculated as mean ± standard deviation (s.d.) values.

### Core flooding experiment

Taking the core-flooding experiment, the potential application of strain SL was evaluated to enhance the oil recovery in reservoirs with low permeability. Artificial core was analyzed for petrophysical features. The core flooding experiment was conducted at 55 °C to simulate the real reservoir temperature. The main experimental steps in this article are listed as follows.

#### Saturation of core with strata water

The artificial core was placed into a core holder and added about 3 MPa of ring pressure. The prepared core was placed under vacuum conditions for 6 h to remove air. The core was then flooded with strata water overnight to ensure its complete saturation. The pore volume (PV) was equal to the volume of strata water stuck in the core.

#### Saturation of core with oil

Crude oil from the Daqing Oilfield was applied in this flooding experiment. The oil, filled in a tank, was placed in a temperature-controlled oven for 2 h in order to sufficiently preheat. Four pore volumes of prepared oil were then pressed into the core at flow rate of 0.2/min to ensure its 100% saturation, following by an aging for 7 days. As oil inserted into the core, the strata water was displaced and discharged from the other end of the core. Original oil saturation was calculated by measuring the PV and the volume of strata water displaced by crude oil.

#### Water flooding and microbial flooding

In the water flooding stage, the strata water was injected into the core until the water cut reached at least 98%. The displaced oil was collected by a test tube to calculate residual oil saturation after water flooding.

Subsequently, 0.5 PV broth of the strain SL was injected into the core at a flow rate of 0.2 ml/min, followed by a 4-day shut-in period. Finally, the water flooding was conducted again to displace the residual oil in the core, until water cut exceeded 98%. The displaced oil was collected again to calculate the additional oil recovery (AOR) with a formula as follows:

2 $${\text{Addtional oil recovery(AOR) = }}\frac{{\text{Oil recovered by secondary water flooding}}}{{\text{Residual oil in core after first water flooding}}} \times 100\%$$

## Results and discussion

### Morphological characterization and bacterial identification

The strain SL is a gram-positive and rod-shaped bacterium. The cell measured has a length of 1.1–1.3 μm and width of 0.6–0.7 μm. Colonies on LB medium appeared to be white, elliptical, convex, rough, and non-transparent, with irregular edges.

**Figure 2:**
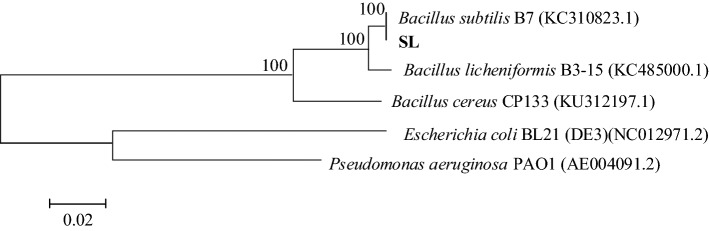
Phylogenetic tree of strain SL based on 16S rDNA sequences of closely related microorganisms.

The results of 16S rDNA sequence analysis show that the sequence similarity between strain SL and *B. subtilis* B7 is up to 99%, indicating a notably high relevance. Phylogenetic tree of the 16S rRDA was constructed by the neighbor-joining method and their close relatives were retrieved from the GenBank database (Fig. 2). Based on the morphological observation and 16S rDNA sequence analysis, strain SL is confirmed to be *Bacillus subtilis*.

### Characterization of produced biosurfactant

**Figure 3:**
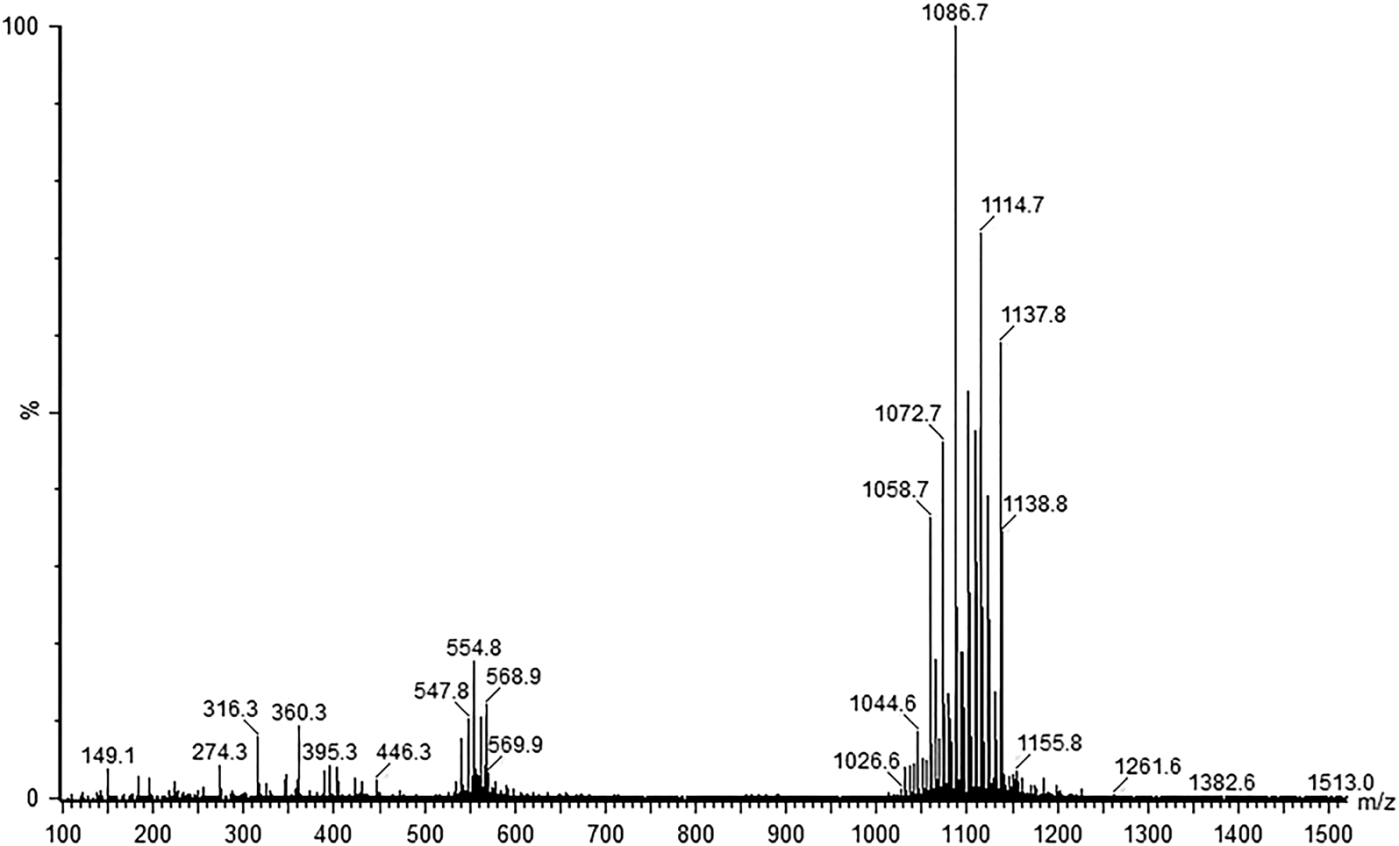
Mass spectrometry analysis of biosurfactant produced by strain SL.

The ESI–MS for biosurfactant produced by strain SL presents the peaks at m/z values, which correspond to the pseudo molecular ions formed from biosurfactant molecules. Four predominant isoforms are identified in the protonated form [M + H] + with m/z at 1044, 1058, 1072, 1084 (Fig. 3). They are constituted of fatty acids varying from C14 to C17. The spectra obtained resembles that of surfactin produced by *B. subtilis* AB2.0^10^. Hence, the biosurfactant produced by strain SL is classified as surfactin. This compound is a lipopeptide that presents a high surface activity and is typically produced by *Bacillus subtilis*.

### Cell growth, surface tension reduction and biosurfactant production

**Figure 4:**
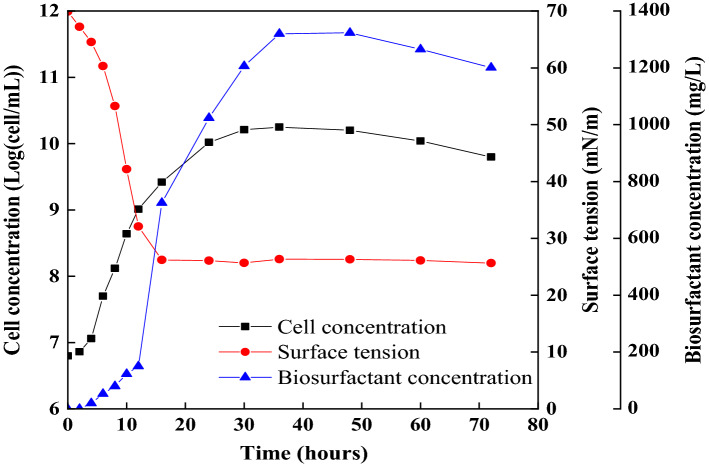
The change of cell concentrations, surface tension, biosurfactant concentration.

Using sucrose as the sole source of carbon, *B. subtilis* SL was studied for its growth and subsequent production of biosurfactant, which is analyzed in terms of cell concentration, biosurfactant concentration and surface tension (Fig. 4). It is observed that the cell growth entered the exponential phase, after a short lag phase. In this phase, the number of bacteria increased exponentially due to the rich nutrition furnished by environment. The sustained growth up to 48 h indicates a stationary phase from 24 to 48 h. The number of bacteria was stable in this stage for the reason that the remains of nutrition could not hold the rapid proliferation of the bacteria. Subsequently, the cell growth declined and entered the death phase (> 48 h). While the death of other bacteria, such as *B. subtilis* AB2.0, *B. pumilus* 2IR and *B. licheniformis*, is after 72h^10,18,19^. Many factors could contribute to this result, such as the depletion of carbohydrates and essential nutrients, accumulation of metabolites, physicochemical and pH variation, etc.

A noticeable decrease in SF was observed during the exponential phase up to 24 h of incubation. The minimum SF value obtained was equal to 25.65 ± 0.64 mN/m during biosurfactant production, which was in accordance with the results observed by Aboelkhair et al.^20^. In addition, the IFT was significantly reduced with the production of biosurfactant and the minimum value can approach 0.95 ± 0.22 mN/m (Data not shown), after 72 h incubation. It has been demonstrated by previous reports that the *B. subtilis* tis capable of producing biosurfactants to diminish the SF and IFT^21,22^. For example, Wang et al. reported that *B. subtilis* can decrease ST and IFT against crude oil to 25.75 ± 0.032 mN/m, and 1.55 ± 0.024 mN/m, respectively^17^.

The biosurfactant production was calculated by oil spreading method from the starting point until 72 h. The observed maximum yield of biosurfactant was 1369 mg/L. The crude yield of biosurfactant was about 1320 mg/L obtained by acid precipitation and solvent extraction. In terms of yield value, the two approaches are not much different. In this section, the yield of crude biosurfactants acquired from previous works (*B. licheniformis*, *B. subtilis*, and *Clostridium *sp.) and our investigation were compared and displayed in Table 1, expecting to obtain a profound understanding and facilitate the development of production in this field. As shown in Table 1, it is obviously denoted that the capacity of *B. subtilis* SL to produce biosurfactant was outstanding and efficient.

**Table 1:** Yield of crude biosurfactant from various microorganisms.

Microorganism	Carbon source	Yield of biosurfactant (mg/L)	References
*B. licheniformis* DS1	Crude oil	400	^23^
*B. subtilis* RI4914	Sucrose	633	^24^
*B. subtilis* DM-04	Crude oil	650	^25^
*B. subtilis* BS-37	Glucose	971	^26^
*B. licheniformis*	Glucose	1000	^19^
*Clostridium *sp. N-4	Sucrose	1000	^15^
*C. albicans*	Glucose	1200	^19^
*B. subtilis* SL	Sucrose	1320	–
*B. subtilis*	Sucrose	1600	^27^
*B. subtilis* B20	Molasses	2290	^28^
*B. subtilis* RSL 2	Crude oil	3500	^21^

### Determination of critical micelle concentration (CMC) and emulsifying capability

**Figure 5:**
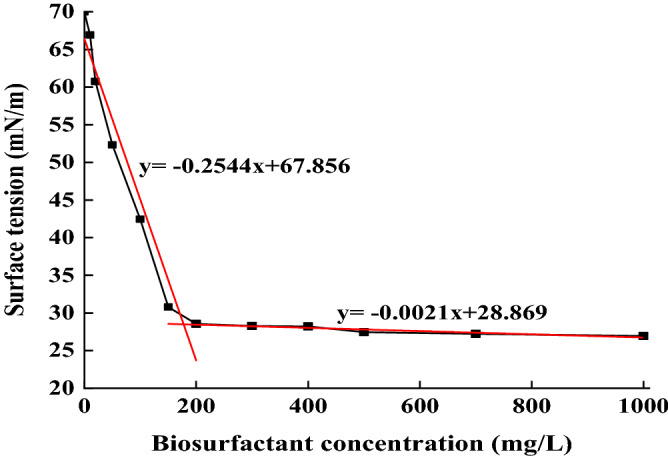
CMC analysis of the crude biosurfactant using a tensiometer.

One of the most essential activities for biosurfactant is the ability to decrease the SF of solution. Initially, the SF decreases gradually with the increase of biosurfactant. Then, when the SF confronts a certain point, the SF ceases decreasing and comes to its nearly stable plateau, which may be ascribed to the saturation of surface by surfactant molecules. Here, this certain point is referred to as critical micelle concentration (CMC). As shown in Fig. 5. the SF exhibits a negative correlation with concentration of crude biosurfactant and the inflection point is found between 150 and 200 mg/L. The lowest CMC value of biosurfactant produced by *B. subtilis* SL is obtained at a concentration of 154 mg/L with a minimum SF value of around 28.68 ± 0.49 mN/m. This result shows that the effective CMC value of the biosurfactant is determined to be 154 mg/L, which is lower than the CMC value of crude biosurfactant extracted from the fermentation broth of *B. subtilis* strain W19 and *B. subtilis* RSL 2 (400 mg/L and 500 mg/L, respectively) ^21,29^. Compared to chemical surfactants like sodium dodecyl sulfate (SDS), the biosurfactant performs better surface activity and lower CMC value^13^. Furthermore, these natural biosurfactants are qualified with low toxicity and superior biodegradability^30^, making them a viable alternative to chemical surfactants.

Except for the SF activity, emulsifying property of biosurfactant is also a principal factor that influences its application in MEOR. Emulsification mediated by the biosurfactants can improve the mobility of crude oil in reservoirs and promote its extraction. The biosurfactant produced by strain SL shows appreciable emulsification activity with crude oil, kerosene, octane and cetane in the experiment, whose emulsification index (EI_24_) is 56%, 67%, 54% and 60%, respectively. The results show the prominence of the strain SL biosurfactant for kerosene.

### Effect of temperature, pH, and salinity on biosurfactant stability

**Figure 6:**
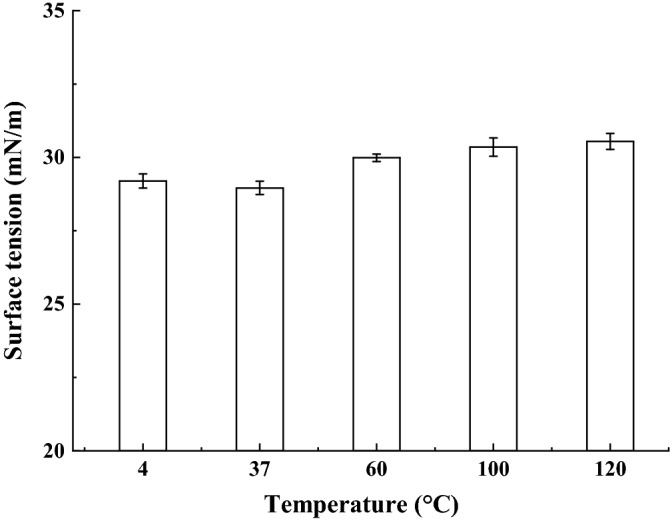
Effect of temperature on biosurfactatnt stability produced by *B. subtilis* SL.

The stability of biosurfactant produced by strain SL was monitored at temperature 4–120 °C, pH 2.0–10.0, and salinity 0–20.0%. To study the surface activity of the surfactant at extreme temperatures, the samples were placed at different temperatures for 24 h (4 °C, 37 °C, 60 °C, 100 °C), even sterilized at 120 °C for 15 min. The surface tension of biosurfactant produced by strain SL remained unchanged when the temperature rose from 4 to 120 °C, revealing its stability in test (Fig. 6). The thermostability of biosurfactants plays an effective role in the application of cosmetics, food, pharmaceutical, petroleum, and bioremediation, where the candidates are required to tolerate extreme harsh temperature conditions.

**Figure 7:**
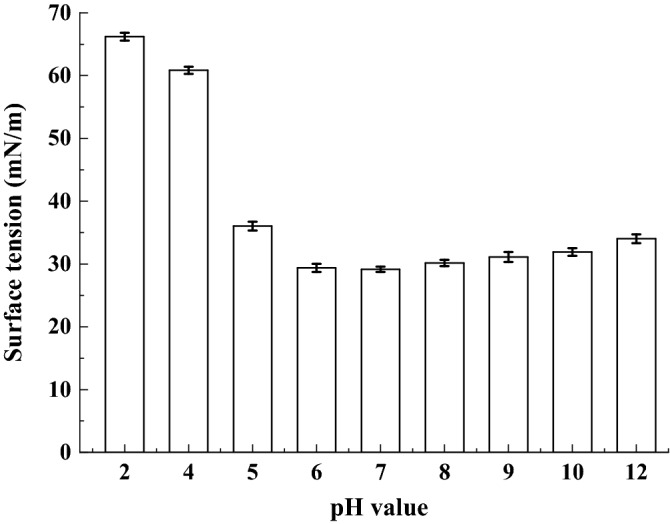
Effect of pH on biosurfactatnt stability produced by *B. subtilis* SL.

The effect of pH on the surface activity of biosurfactant produced by strain SL is depicted in Fig. 7. It can be seen that the biosurfactant possesses superior surface activity in pH range of 5.0–12.0. However, when the pH is lower than 4, the biosurfactant begins to precipitate and its surface activity was comparatively impaired.

**Figure 8:**
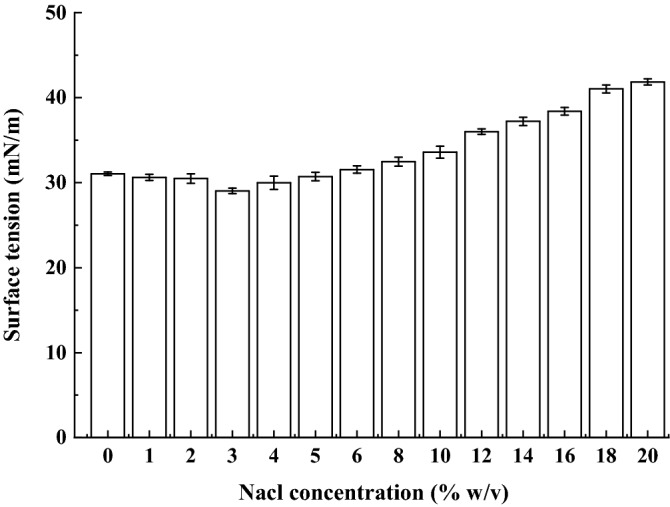
Effect of NaCl concentration on biosurfactatnt stability produced by *B. subtilis* SL.

The effect of salinity on the surface activity of biosurfactant produced by strain SL is shown in Fig. 8. When the concentration of NaCl varies between 0% and 10.0% (w/v), the surface tension remains around 30 mN/m and the activity of strain SL products are virtually unaffected. However, when the concentration exceeds this range, the surface tension is improved significantly. Additionally, it is apparent that the biosurfactants exhibit superior salinity tolerance to chemical surfactants which normally deactivate with salinity ranged in 2.0–3.0% (w/v) ^31^.

Collectively, the biosurfactant produced by strain SL shows splendid surface activity at pH 5.0–12.0, salinity 0–10.0% (w/v), and even at 120 °C. Similarly, Wang et al. proposed that the biosurfactant are stable at 30–100 °C and 0.5–25.0% (w/v) of salinity^17^. Aboelkhair et al. reported that the biosurfactant exhibited super stability against high salt and temperature concentration, even at NaCl concentration of 20.0% (w/v) and temperature of 120°C^20^. The stability of biosurfactants at broad range of temperature, pH and salinity is supported by many reports. In conclusion, the biosurfactants produced by strain SL are capable to tolerate the harsh conditions and maintain good surface activity, indicating as a suitable candidate for MEOR.

### Enhanced oil recovery in low permeability reservoirs by strain SL

The crude oil used in this study was provided by Daqing Oilfield, whose viscosity and density is 17.2 mPa·s and 0.8737 g/cm^3^ respectively. The strata water adopted in core flooding experiments is NaHCO_3_ solution with a total mineralization of approximately 8158 mg/L^32^. The length, diameter, air permeability, and water permeability of core sample A and B are measured to be 81.0 mm, 25.0 mm, 46.64mD, 11.46mD, and 80.5 mm, 25.0 mm, 48.61mD, 13.98mD, respectively.

**Figure 9:**
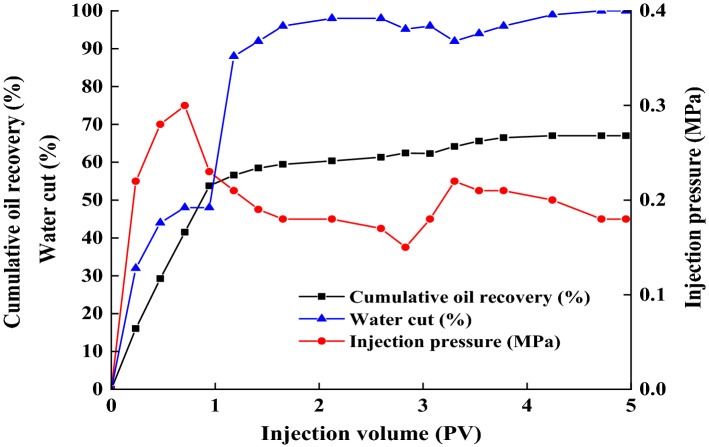
Core A: water ratio, oil recovery ratio and injection pressure during process of core flooding tests.

As presented in Fig. 9, the variation of water cut, injection pressure, and cumulative recovery of core A during the entire flooding process are displayed. It is observed that at the early stage of water flooding, the dramatically increased water cut was accompanied with a descending displacement pressure, and the oil recovery rate reached the plateau of the water flooding stage with injection around 1.0 PV. Then, the water flooding was proceedings until the injected pore volume was approached 2.6 PV together with the water cut about 98%. In this process, there is no crude oil escaping virtually. Finally, the oil recovery after water flooding accomplished 61.63% of the original oil in place. This mean, of course, that there is still about 36% of the crude oil trapping in the core. Subsequently, 0.5 PV of the strain SL broth was injected into the core which was shut in and incubated at 55 °C for another 4 days. The shut-in period was followed by secondary water flooding. During this stage, after injecting 1.0–2.0 PV, the water cut reached at least 98%, and about 5.66% of additional oil over the first water flooding was recovered after the secondary water flooding. During the experiment, it was not difficult to see that the injection pressure of core A increased by 23.52% and the water cut decreased by 6.12%, after the shut-in period. This may be attribute to multiple factors that are discussed in the following context. Firstly, the biosurfactant produced by strain SL can reduce oil–water interfacial tension and emulsify crude oil. Secondly, *B. subtilis* can degrade a variety of components in crude oil. Whether the function of biosurfactant or biodegradation, both of them work effectively to mobilize residual crude oil in the core, and hence reduce the water cut of the recovered fluid. Additionally, some other factors might influence the pressure and water cut. For example, gases such as carbon dioxide produced by microbial respiration during shut-in period may increase the pressure in cores. Furthermore, the biomass may affect the pressure and water cut as well. The adsorption and retention of biomass in the core pore throat could efficiently plug the high permeability region to mobilize the residual oil in the low permeability area. Bi. et al. have reported that microbial biomass performed better in the blocking and regulating cores with lower permeability^33^.

In term of core B, it almost complies with the same law as core A. Specifically, the oil recovery after the primary water flooding and the secondary water flooding is equal to 62.74% and 68.13%, triggering an ultimate enhanced oil recovery at 5.39%. Several researchers have reported the potential application of MEOR in low permeability reservoirs. Xiao et al. utilized the glass micro-models with low permeability to conduct indigenous microbial enhanced oil recovery experiments and gained an additional 6.9% of crude oil^7^. Sarafzadeh et al. reported that biosurfactant producing *Bacillus stearothermophilus* SUCPM#14 is promising for MEOR in low permeability reservoirs. They have performed core flooding experiment by employing carbonated cores with low permeability, and obtained an additional 4.21% (without shut-in period) and 5.80% (shut-in a week) of crude oil^34^. The higher enhanced recovery (5.66%) in low permeability core flooding tests enables strain *B. subtilis* SL to be an excellent candidate for microbial enhanced oil recovery in low permeability reservoirs.

## Conclusion

This study presented here confirm the potential of *B. subtilis* SL for MEOR in low-permeability reservoirs. The biosurfactant exhibits the ability to reduce the SF to 28.68 mN/m at CMC concentration, and shows a superior emulsification on diesel and cetane. Meanwhile the biosurfactant has good stability over a wide range of pH, high temperature and high salinity. From core flooding experiment, the *B. subtilis* SL can extract an additional 5.66% of crude oil from low permeability cores. However, it is still necessary to carry out additional field trails to demonstrate the applicability of *B. subtilis* SL and its biosurfactant on enhancing the oil recovery of low permeability reservoirs (Supplementary Information).

## Supplementary Information

Supplementary Information.
